# Involvement of dendritic cells in autoimmune diseases in children

**DOI:** 10.1186/1546-0096-5-16

**Published:** 2007-07-11

**Authors:** Consuelo M López de Padilla, Ann M Reed

**Affiliations:** 1 Division of Rheumatology, Departments of Medicine, Pediatrics, and Immunology, Mayo Clinic College of Medicine, Rochester, MN, USA

## Abstract

Dendritic cells (DCs) are professional antigen-presenting cells that are specialized in the uptake of antigens and their transport from peripheral tissues to the lymphoid organs. Over the last decades, the properties of DCs have been intensely studied and much knowledge has been gained about the role of DCs in various diseases and health conditions where the immune system is involved, particularly in cancer and autoimmune disorders. Emerging clues in autoimmune diseases, suggest that dendritic cell dysregulation might be involved in the development of various autoimmune disorders in both adults and children. However, studies investigating a possible contribution of DCs in autoimmune diseases in the pediatric population alone are scanty. The purpose of this review is to give a general overview of the current literature on the relevance of dendritic cells in the most common autoimmune conditions of childhood.

## Background

Autoimmune disorders are chronic disabling disorders in which underlying defects in the immune response resulting in an inappropriate response to self-tissues. Collectively, autoimmune diseases are thought to affect approximately 14–22 million people in the United States, according to the American Autoimmune Related Diseases Association (AARDA) representing greater than 80 unique disorders [1], which affect preferentially women.

Autoimmune disorders in children are less common but important to diagnosis, as immunomodulating therapy can prevent considerable mobility and mortality, and improve the survival of many of these patients. Autoimmune diseases are characterized by the loss of tolerance against self-determinants, activation of autoreactive lymphocytes and/or autoantibodies, with pathological damage that is organ specific or systemic.

A clear understanding of the mechanisms by which autoimmune responses are triggered leading to the activation and/or induction of autoreactive lymphocytes and to the breakdown of immunological self-tolerance are not yet fully understood. Currently, multiple lines of evidence indicate that DCs may also participate in the onset of autoimmune diseases. [2-5]. Animal models show the transfer of DCs isolated from donors with acute autoimmune disease or propagated in vitro under conditions that induce maturation, generates a strong T helper (Th)-1 response, eventually culminating in autoimmune disease [6]. For instance, one of the hallmarks of systemic autoimmune diseases, such as systemic lupus erythematosus (SLE), is the immune response to nuclear autoantigens. Several studies have proposed that DCs may acquire the nuclear autoantigens from the apoptotic cells to initiate the systemic autoimmune responses [7,8]. On the other hand, experimental models of diabetes in mice suggest that DCs may trigger autoimmunity by initiating a local or systemic response to an infective agent [9]. Increased numbers of DCs have been described in chronic arthritis synovial membrane and fluids [10-12]. However, there have been a limited number of studies investigating the role of DCs in the immunopathogenesis of autoimmune disorders in childhood.

We think that, a better understanding of unique pathogenic involvement of DCs in autoimmune diseases will hopefully lead to the development of better, more targeted and less toxic therapies and could contribute to improve their outcome. Therefore, targeted modulation of DC function may open new treatment options aiming at alleviating DC-driven autoimmune responses. In this review, an attempt has been made to summarize recent data on DCs physiology in autoimmune diseases in the pediatric population.

### Dendritic cells: key cells in autoimmune diseases?

Briefly, DCs are a population of bone marrow-derived leukocytes that efficiently link the innate and adaptive immune systems and play a crucial part in initiating, amplifying and controlling the immune response to pathogenic microorganisms [13]. DCs reside in and traffic through non-lymphoid peripheral tissues where they develop to a stage referred to as immature DCs, continuously surveying the environment for invading microorganisms. These immature DCs (iDCs) are characterized by high capability for antigen capture and processing, but low antigen presentation and T cell stimulatory capabilities. DCs in the periphery are triggered by exposure to microbial agents such as lypopolysaccharide (LPS) or inflammatory mediators such as tumor necrosis factor (TNF-α) and interleukin-1 (IL-1) are induced to enter in a maturation process [14,15].

During their conversion from immature to mature cells, DCs undergo a number of phenotypic and functional changes. They lose their ability to capture antigen and transform into efficient antigen presenting cells (APCs). The process of DC maturation is characterized by an increased surface expression of major histocompatibility complex (MHC) class I and class II, up-regulation of T-cell costimulatory molecules such as CD80, CD86 and CD40, secretion of chemokines and cytokines, surface expression of adhesion molecules and chemokines receptors [16]. Chemokine receptors promote DC migration out of nonlymphoid tissues into the blood or afferent lymphoid vessels, as well as enhanced T cells stimulation. These migratory cells reach secondary lymphoid organs where they home to the T cell areas, and interact with, stimulate and direct primary T- and B- lymphocytes responses. Maturation of DCs modifies expression of chemokines receptors and adhesion molecules, causing migration from the periphery to the T cell zone of secondary organs. [17,18]

The types of T cell-mediated immune responses induced can vary depending on the specific DC lineage (myeloid vs. lymphoid) and maturation stage in addition to the activation signals received from the surrounding microenvironment. During the primary immune responses, the DC subset is a critical determinant for polarizing naive T cells into Th1 or Th2 cells [19]. Studies over the last decade have demonstrated that the distinct subsets of human DCs (myeloid and plasmacytoid DCs a form of lymphoid DC) induce the different profiles of T cells responses [13,15,20]. For example, studies have shown that myeloid DCs (mDCs) produce a large amount of IL-12 and preferentially induce Th1 development, whereas plasmacytoid DC (pDCs) secrete lower amounts of IL-12 and primarily elicit Th2 development [21-24]. On the other hand, other studies have suggested that functional differences between DCs in guiding T cell responses might depend on not only their lineage but also the microenvironment of cytokines and/or inflammatory mediators in the primary immune response [22,24].

### Role of dendritic cells in children autoimmune diseases

#### Dendritic cells in children lupus

Systemic lupus erythematosus (SLE) is an autoimmune disease characterized by polyclonal B-cell activation, the production of antinuclear antibodies (ANAs) and systemic inflammatory injury of multiple organs. SLE constitutes up to 10 percent of the new diagnosis of systemic rheumatic diseases in childhood [25]. The pathogenesis of SLE is correlated with both genetic predisposition and environmental influences. The contribution of these two factors may differ between individuals, but the resulting malfunctions in the immune system and the production of autoantibodies plays a pivotal role in the pathogenesis of SLE; however, the mechanisms of how this occurs in SLE pathogenesis remains unclear. Lupus has been considered mainly as B-cell disease resulting from altered T-cell and B-cell interactions [26,27]. The immune system abnormalities seen in patients with lupus are diverse and include CD4+ autoreactive T cells that are specific for ubiquitous self-peptides and provide pathogenic B cell help, ANA-producing autoreactive B cells, alterations in Th1 and/or Th2 lymphocyte functions resulting in production of cytokines that up-regulate autoantibody production by B cell, promote immune complex formation which determine different subsets of the disease [26,28,29].

The mechanisms by which an autoimmune response is triggered and activation of autoreactive lymphocytes is initiated and maintained in SLE are not fully understood. Different groups have proposed that T cell abnormalities in SLE could be induced or promoted, at least in part, by alterations in DCs phenotype and function, because these are key regulators of the immune system [30-32]. Naïve T lymphocytes circulate through the blood and secondary lymph organs (SLOs), where they scan for antigen-histocompatibility complex molecule displayed on dendritic cells. If accompanied by appropriate co-signals, naïve T cell-DC interaction induces T cell clonal expansion and promotes migration either to B cell area to assist in antibody production or out of the lymphoid tissue to sites of inflammation [33]. Moreover, DCs appear to be the only class of antigen presenting cells that have the capacity to stimulate the expansion of naïve T cells and thereby initiate primary immune responses [15,34,35]. DCs can also generate regulatory T cells that suppress activated T cells, a function of probable importance in autoimmunity [36].

Type I interferons (IFNs) are produced in response to viral and non-viral infections and may be induced during T cell-DC interactions in the absence of infecting agents [37]. Type I IFNs have been shown to act as a maturation factor for DCs and to increase DC maturation [38,39]. Patients with SLE have increased blood levels of IFN-α, which correlate to disease activity [32,40-42]. In addition, drug-induced lupus has been reported in hepatitis C or cancer patients treated with recombinant IFN-α [43]. This evidence suggests a significant role to the pDCs, the major source of IFN-α in the blood, in the immunopathogenesis of SLE. Palucka *et al*. [44] have been the first group to identify how the interaction between lymphoid and myeloid DCs might play an important role initiating the immune response in SLE patients. They linked a hyperactivated secretion of IFN-α by DCs to the immunological dysfunction observed in children with SLE. In brief, serum from children with SLE induced monocytes to differentiate into DCs. Furthermore, they found that DCs are stimulated to proliferate when peripheral mononuclear cells (PBMCs) from normal donors were cultured with the serum of pediatric lupus patients. IFN-α was identified as the cytokine responsible for this effect and, as seen in other reports, pDCs were the major source of interferon in the blood of these patients. From these findings, they have formulated a hypothesis that explains the activation and pivotal action of the type I IFN system in the development of SLE. It is based on the activation of immature myeloid dendritic cells through type I IFNα/β. IFN-matured mDCs are proposed to efficiently capture autoantigens (apoptotic cells and nuclesomes) and then present the antigens to CD4+ T cells which initiate the expansion of autoreactive T cells. The mDCs together with pDCs could help to expand autoreactive B cells, followed subsequently by autoantibody production. This abnormal immune reaction could finally lead the immune system of lupus patients to attack their own tissues with the subsequent injury to various organ systems, including skin, kidney and central nervous system [44-46]. Ronnblom et al. implicated pDCs and IFN-α in adults with SLE [42,47]. Interesting others investigators have demonstrated in SLE subjects that circulating immune complexes ((ICs), in addition to INF-α, activates DCs and can induce cytokines and chemokines implicated in SLE pathogenesis. For instance, the local production of the chemokines IL-8, MCP-1, and RANTES has been noted in inflamed joints of SLE patients, and serum levels of MCP-1 and IP-10 are higher in SLE patients than in controls [48,49]. Means et al. demonstrated that anti-DNA autoantibody ICs were able to activate DCs through a cooperative interaction between Toll-like receptor 9 (TLR9) and FcγRIIa (CD32) [50].

These findings may provide a framework for developing better lupus therapies that the currently available, however more research is needed to clarify the exact role of DCs in lupus pathogenesis.

#### DCs in Juvenile Dermatomyositis

**Figure 1:**
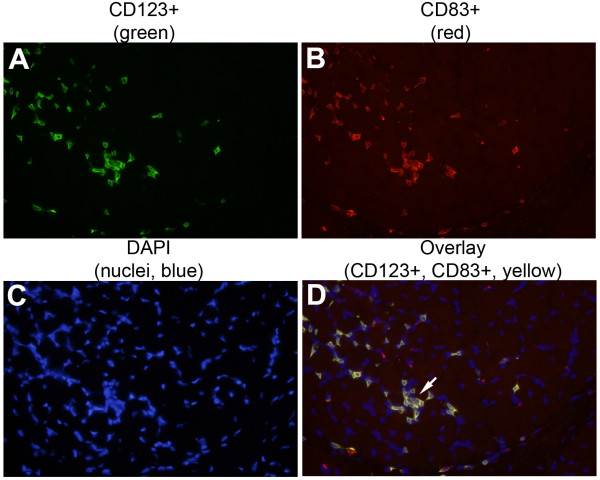
**Activated pDCs in JDM muscle**. JDM muscle samples were stained for CD123 (green fluorescent avidin), CD83 (Texas Red), and the nucleus (DAPI). Most of the pDCs in the JDM muscle were activated (CD123+ CD83+, yellow in overlay). Magnification: × 40.

**Figure 2:**
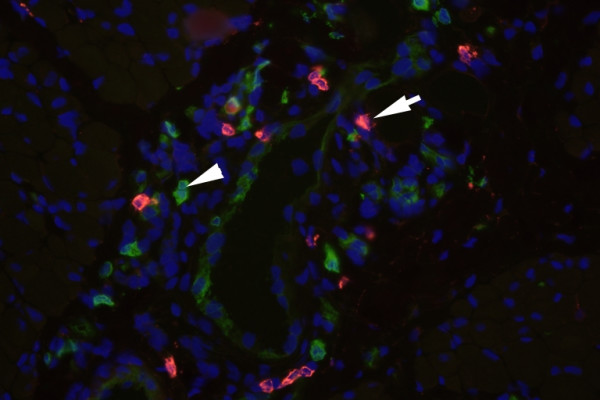
**Architecture and cellular composition of inflammatory infiltrates in JDM muscle**. pDCs (arrow heads denote pDC aggregates) were localized in close proximity to B-cells (arrows denote CD20+ B cells) and T-cells (not shown). Magnification: × 40

Juvenile DM is the most common of the idiopathic inflammatory myopathies in children. It is considered an autoimmune disease of etiology and pathogenesis relatively unknown. The inflammatory lesions consist of perivascular, perifasicular and intradermal lymphocytes infiltrates. The perivascular infiltrates are composed mainly of CD4+ T lymphocytes, B lymphocytes, macrophages [51]. While the exact triggers of the immune response in children with juvenile DM have not been identified, recent studies have attempted to elucidate the targets of such abnormal immune responses. The presence of T cells indicates an ongoing immune response requiring the presence of APCs. Emerging data suggest that DCs, the most potent APCs, may be involved in the immunopathogenesis of the idiopathic inflammatory myopathies (IIM). Mature DCs have been detected in dermatomyositis and polymyositis muscle tissue, predominantly located in perivascular infiltrates and surrounded muscle fibers [52]. Recently, our laboratory demonstrated that pDCs populate muscle lesions of patients with new-onset Juvenile DM [53]. We compared samples of muscle tissue from children with Juvenile DM to patients with non-inflammatory muscle disorders using triple immunofluorescence analysis (Figure 1). We found that pDCs populate muscle lesions of patients with new onset JDM, the majorities of these pDCs were mature as indicated by high CD83 expression and they were localized in close proximity to T cells and B cells in the perimysium and perivascular areas (Figure 2). Since such cellular infiltrates contained a large proportion of T cells, presence of mature pDCs is consistent with the idea that these infiltrates are foci of inflammation. Furthermore, we also found that DC-derived chemokines CCL21, CCL19 and CXCL12 ("lymphoid" chemokines), which have been demonstrated to be up regulated in ectopic secondary lymphoid organs at sites of chronic inflammation [54-56], were over-expressed within organized infiltrates in Juvenile DM muscle lesions. The simultaneous presence of DCs, T-and B-cells and the chemokine profile found in the inflamed muscle tissue of these patients suggests that interactions among these cells types might be instrumental in the local induction and maintenance of neo-lymphoid genesis in JDM muscle.

Recent advances in the study of patterns of gene expression with the use of microarray technology, has provide new insights into the study of role of pDCs in the immunopathogenesis of adult and Juvenile DM. A gene expression profile identified by microarray analysis and consistent with IFN-mediated gene transcription in inflammatory myositis was first reported by Tezak and colleagues in a study of muscle biopsies from JDM and age-matched controls [57]. They compared the gene expression profile data from children with untreated Juvenile DM, positive for the DQA1*0501 allele, with data from children with Duchene muscular dystrophy and healthy pediatric controls, as well as profiles reflective of intracellular antiviral response by using Affymetrix HuFL Gene Chips. They found profound dysregulation in muscle biopsies from children with Juvenile DM, most of the dysregulated genes being interferon-inducible genes. This pattern of gene expression found was consistent with an of IFN α/β transcription cascade seen in an *in vitro *viral resistance model. These findings gave additional support to the hypothesis that the pathogenesis of Juvenile DM is a response to an infection agent, particularly since transcription of IFN-inducible genes is a hallmark of the host defense mechanism against infection. From this profiling data, the authors hypothesized a model of disease pathogenesis that involves a repetitive cycle of muscle injury in which both INF-α/β and INF-γ cascades lead to muscle ischemia and increased production of TNF-α and nitric oxide, which in turn, interact with the immune response cascades in the endothelium and with infiltrating T and pDCs, thus exacerbating the IFN-induced processes. In a recent work from our laboratory [58], we investigated patterns of gene expression profiling of peripheral blood cells from adult and Juvenile DM and healthy controls by using as well microarray analysis. We studied the expression levels of 314 IFN-inducible genes between adult and Juvenile DM and healthy controls. We found that in the most of DM patients 93 out of 314 genes examined were overexpressed in DM patients, which correlated with disease activity. The particular interest, we noted the increased expression of a number of genes involved in muscle function, mitochondrial/oxidative phosphorylation and immune function. Many genes previously demonstrated by other investigators as overexpressed in other autoimmune diseases such as SLE [59,60], Sjögren's syndrome [61] and inflammatory myopathies [62] were similarly increased in our data. Together, our findings suggested that pDCs are an important source of IFN-α/β in adult and childhood DM and may therefore be of pathogenic importance, and even more relevant the IFN-profile found could points towards the type I IFN-α/β pathway as a candidate pathway for DM susceptibility genes.

#### DCs in Juvenile Inflammatory Arthritis

Juvenile arthritis is a group of chronic inflammatory diseases in children collectively known as Juvenile Idiopathic Arthritis (JIA). It is the most common chronic autoimmune arthropathies of childhood and an important cause of disability. JIA is characterized by a persistent inflammatory infiltrate within the synovial layer of the joints. The findings of T cells and chronic synovial inflammation have indicated a possible cellular-mediated immune response. However, the presences of immune complexes, complement, and inflammation in JIA have indicated a possible B cells hyperreactivity [63]. T cells within the hypertrophied synovium are activated, oligoclonal, and has a bias towards a T helper (Th1) phenotype in oligoarticular and polyarticular JIA. The immune response identified by the induction of Th1/Th2 cells may play an important role in the immunopathogenesis of JIA [64,65]. However, it is unclear what causes clonal inflammatory lymphocytes within the joints of these patients. One mechanism could be through signals received from APCs such as DCs. DCs may contribute to the synovitis by homing T cells as well as by the induction of cytokines belonging to both Th1/Th2 cells. Varsani et al [66] have recently found an increased number of cells with DC morphology, in the joint of children with JIA. Specifically, they examined the expression of the receptor activator of NF-κB (RANK), a TNF receptor-like surface protein derived from DCs, and its ligand (RANKL). The RANK/RANKL pathway is critical in bone erosion in conditions such as rheumatoid arthritis [67] and is involved in DC-T cell interactions. They investigated the expression of RANK/RANKL in paired samples of peripheral blood (PB) and synovial fluid (SF) from JIA patients, as well as in monocyte-derived dendritic cells by the reverse transcriptase-polymerase chain reaction and flow cytometry. They determined that RANK and RANKL were overexpressed in cells from both PB and SF compartments of children with JIA as compared to controls. Interesting, a large number of RANK+ synovial cells had the phenotype of mature myeloid dendritic cells. Mature DC may play a central role in the presentation of antigens to T cells and the production of inflammatory cytokines. These RANK+ cells also expressed the DC-specific adhesion receptor DC-SIGN (CD209), which supports rolling of DC-SIGN+ cells on the vascular ligand ICAM-2 under shear flow, a prerequisite for DC emigration from the blood into the peripheral tissues. Another novel finding of this study was that the majority of RANK+ DC-SIGN+ cells also expressed high levels of the costimulatory molecule CD86; the binding of this protein with CD28 antigen at the cell surface of T cells could be a costimulatory signal for activation of the T-cell in JIA synovium. Taken together, these findings suggest that RANK+ DCs in children with JIA might contribute to persistence and expansion of particular T cell subset within inflamed joint of JIA patients.

Similarly, Palucka et al. [68] implicated INF-α production by pDCs in the immunopathogenesis of JIA. They have proposed that the induction of autoimmunity to nuclear antigens during anti-TFN-α therapy appears to be induced by IFN-α. They found that children with systemic onset JIA (SoJIA) who did received anti-TNF therapy displayed over-expression levels of IFN-α-regulated genes in their blood leukocytes compared with patients without anti-TNF therapy. In addition, they found that the IFN-α production by pDCs generated from CD34+ hematopoietic progenitors exposed to influenza virus was inhibited by TNF. These findings led them to propose that the neutralization of endogenous INF during anti-TNF therapy might further switch the TNF/INF-α balance to a sustained IFN-α secretion by pDCs with a well-known pathogenic effect, and therefore a possible explanation to the lupus-like symptoms develop during TNF blockade.

#### Role of dendritic cells in vasculities

Recent studies have investigated the participation of DCs in the pathogenesis of various vascular inflammatory diseases such as Giant Cell Arteritis (GCA) [69], Takayasu's Arteritis [70] and atherosclerosis, which is now emerging as an inflammatory syndrome [71]. Weyand et al [69,72] have intensely investigated the mechanisms involved in the persistence and expansion of DCs in the vessel wall of medium-sized arteries from patients affected by GCA, the most common form of systemic vasculities in human. They found a number increased of mature (activated) DCs positioned in the adventitia-media border of these vessels (adventitial DCs), which were equipped molecularly to contribute to vascular lesion. Moreover, these DCs might be influenced by antigen-specific signaling from T cells, which may extend and amplify DC antigen capabilities, especially for the stimulation of cytotoxic responses.

The involvement of DCs in the immunopathogenesis of Kawasaki disease, a multisystem vasculities associated with cardiac complications in children, has been suggested by Yilmaz and Colleagues [73]. They studied the participation of DCs in the immunological abnormalities seen in the coronary lesions of Kawasaki's patients. They found an increased number of mature myeloid DCs (S100+, Fascin+, HLA-DR+, CD83+) in the adventitia of coronary arteries. Interesting, they observed mature DCs with high HLA-DR expression in close proximity to T cells evoking DC-T cell immunological synapse formation, which suggested that mature arterial myeloid DCs might be activating T cells in situ and may be a significant factor in the pathogenesis of coronary arteritis in KD.

Aberrant DC function has also been implicated in other, less prevalent autoimmune diseases in childhood such as cutaneous scleroderma [74]. In addition, the pathogenic role of DCs in the pathogenesis of Type I Diabetes has been recently suggested in several studies [75,76]; however the analysis of impact of DC malfunction in this disease has not been considered in this review.

## Conclusion

Based on the evidence obtained from various autoimmune diseases described here, it is highly suggestive that local or recruited DCs play a fundamental role in both induction and maintenance of these disorders. Moreover, direct analysis of DC phenotypes and DC-T cell interactions is several autoimmune diseases bring light on the pathogenesis of these autoimmune disorders. We must take into account the delicate balance between immunity and autoimmunity and the pivotal role DCs play in this equilibrium. Future therapeutic regimes used to fight autoimmune disease need to consider therapeutic options based on specific modulation of pathogenic DCs that induce and sustain autoimmune inflammation, which could be curative or be able to induce long term remissions.

## Abbreviations

Dendritic Cells, DCs; immature DCs, iDCs; Antigen presenting cells, APCs;

myeloid DCs, mDCs; plasmacytoid DC, pDCs; Systemic lupus erythematosus, SLE;

Interferons, IFNs; Juvenile Dermatomyositis, Juvenile DM; Juvenile Idiopathic Arthritis, JIA.

## Competing interests

The author(s) declare that they have no competing interests.

## Authors' contributions

In this review both authors (CLP and AMR) participating in searching, analysis and summarized the current information on the involvement of dendritic cells in autoimmune diseases in children and manuscript preparation.
